# Social Behaviors Associated With a Positive COVID-19 Test Result

**DOI:** 10.7759/cureus.13064

**Published:** 2021-02-01

**Authors:** Sidra L Speaker, Christine M Doherty, Elizabeth Pfoh, Aaron Dunn, Bryan Hair, Lynn Daboul, Victoria Shaker, Michael Rothberg

**Affiliations:** 1 Internal Medicine, Cleveland Clinic Lerner College of Medicine of Case Western Reserve University, Cleveland, USA; 2 Center for Value Based Care Research, Cleveland Clinic, Cleveland, USA

**Keywords:** covid-19, sars-cov-2, infectious diseases epidemiology, contact tracing, coronavirus quarantine, social distancing, survey research, social and behavioral epidemiology, covid-19 transmission, public behavioral health

## Abstract

Objective

To compare the social behaviors of individuals who were tested positive for COVID-19 relative to non-infected individuals.

Methods

We sent COVID positive cases and age/gender-matched controls a survey regarding their social behaviors via MyChart (online patient portal). We called cases if they did not complete the electronic survey within two days. Data were collected from May to June 2020. Survey responses for cases without close contact and controls were compared using Pearson chi-square or Fisher’s exact tests as appropriate.

Results

A total of 339 participants completed the survey (113 cases, 226 controls); 45 (40%) cases had known contact with COVID-19. Cases were more likely to have recently traveled (4% vs. 0%, p = 0.01) or to work outside the home (40% vs. 25%, p = 0.02). There was no difference in the rates of attending private or public gatherings, mask/glove use, hand-washing, cleaning surfaces, and cleaning mail/groceries between cases and controls.

Conclusions

Sixty percent of cases had no known contact with COVID-19, indicating ongoing community transmission and underlining the importance of contact tracing. The greater percentage of cases who work outside the home provides further evidence for social distancing and remote telework when possible.

## Introduction

SARS-CoV-2 has infected >61 million individuals worldwide, with 1.4 million deaths as of December 1, 2020 [1]. SARS-CoV-2 is highly transmissible, with an estimated reproductive number (R0) of 2, approximately double that of influenza [2]. To mitigate the spread of COVID-19, the disease caused by SARS-CoV-2, there has been the widespread promotion of public health measures. These measures, developed from experiences with other coronavirus outbreaks such as SARS, include isolation, quarantine, social distancing, and community containment. Isolation, the separation of symptomatic-infected individuals from non-infected individuals, works best for diseases with a short incubation period. COVID-19 has a long incubation period and a substantial portion of transmission may occur from asymptomatic or pre-symptomatic individuals [3]. Quarantine, restriction of movement of exposed individuals for the duration of the incubation period, is most useful when contacts of infected individuals can be readily traced. Social distancing involves limiting interactions among community members to decrease community spread. Finally, community containment involves restricting movements in entire geographical areas [4].

Population-based studies provide evidence that these measures have slowed the spread of COVID-19. In China, there was a significant reduction in cases following strict community containment [5]. In four United States cities (New York City, Seattle, New Orleans, and San Francisco), new cases declined following the implementation of isolation, quarantining, and social distancing [6].

Less is known about the impact of specific recommendations, such as working from home, outdoor exercise, and disinfecting groceries/packages on the spread of the novel coronavirus. In China, suboptimal hand hygiene was associated with COVID-19 infection among healthcare workers [7]. It is important to identify which public health recommendations are most effective to support adherence and mitigate the psychosocial and economic impacts of unnecessary recommendations. In this case-control study, we describe patterns of COVID-19 prevention behavior among individuals in Ohio and Florida and differences between patients testing positive for COVID-19 versus uninfected patients. This article is available as a preprint [Speaker S, Doherty CM, Pfoh ER, et al. Social Behaviors Associated with a Positive COVID-19 Test Result. medRxiv. Published online January 1, 2020:2020.08.04.20168450. doi:10.1101/2020.08.04.20168450].

## Materials and methods

We surveyed adults with a COVID-19 PCR positive result from our institutional COVID-19 registry and gender- and age-matched controls from the Electronic Health Record (EHR) such that responses had approximately a 2:1 ratio. We sent all patients a 14-question survey regarding protective behaviors via MyChart, Cleveland Clinic’s online patient portal. We developed the survey by reviewing surveys of past epidemics [8,9] and the PhenX toolkit [10]. Based on expert opinion and CDC guidelines, we included questions on contact with a person known to be infected with COVID-19, social distancing behaviors, and hygiene behaviors. The survey was pilot-tested for clarity. Survey responses were stored in RedCap. We sent surveys to cases on the day of or the day following their first positive COVID-19 PCR test result, and to controls weekly. If cases did not complete the electronic survey within two days, we contacted them to administer it by phone. We excluded patients if they were non-English speaking, had dementia, resided in a skilled nursing facility, were currently hospitalized, or were not active on MyChart. Demographic data were extracted from the EHR from May 12, 2020 to June 22, 2020. After identifying individuals who reported close contact with a COVID positive individual, we compared the behavior of remaining cases and controls using Mann-Whitney U or Student’s t-tests (numeric variables), and Pearson chi-square or Fisher’s exact tests (categorical variables) as appropriate. Statistical analyses were conducted using SAS (SAS Institute, Cary, NC) Studio v.9.4 and R Studio v.3.6.3.

## Results

A total of 113 cases and 226 controls completed part or all of the survey (response rate 9%), with 45 (40%) cases reporting having a close contact, compared to <1% of the controls. The results were analyzed comparing patients with no known close contact. The median age was 54 and 63% were female. 

A greater percentage of cases had someone in their residence who provided patient care (21% vs. 10%, p = 0.02). They were also more likely to live with someone experiencing upper respiratory tract symptoms within the past week (22% vs. 3%, p < 0.001). Cases were more likely to work outside the home (40% vs. 25%, p = 0.02), to interact with more than 10 people in their daily work (35% vs. 18%, p = 0.002), and to have traveled via airplane (4% vs. 0%, p = 0.01) or RideShare/Taxi (4% vs. 0.4%, p = 0.04).

A greater percentage of cases were placed in isolation or quarantine (25% vs. 4%, p < 0.001). Cases were less likely to have gone outside to walk, hike, or exercise (52% vs. 88%, p < 0.001).

There were no differences between cases and controls in the rates of attending gatherings, mask/glove use, hand-washing, cleaning surfaces, and cleaning mail/groceries; and no difference in self-reported medical comorbidities (Table 1). 

**Table 1:** Survey Responses Among Cases and Controls *Patients who had a known close contact were excluded from remaining analyses (N = 46) †In the control version this reads: “In the last seven days, I have” Results reported as median (IQR) or N (%)

Survey Question	Cases N = 113	Controls N = 226	P-value
Close contact with someone who has COVID*	45 (40)	1 (0.4)	<0.001
	Cases N = 68	Controls N = 225	
Age	52 (35 -62)	55 (39-70)	0.06
Gender - female	41 (60)	143 (64)	0.44
Number of people living in the home	2 (2-4)	2 (2-4)	0.903
Someone in the home had URI symptoms in past week	15 (22)	7 (3)	<0.001
Someone in the home provides patient care	14 (21)	22 (10)	0.02
In the seven days prior to my test^†^, I have			
Gone to a friend, neighbor, or relative's residence	5 (8)	26 (12)	0.34
Attended a public gathering (more than 10 people)	7 (10)	27 (12)	0.81
Sought care from a hospital or healthcare facility	15 (22)	40 (18)	0.40
Been placed in isolation or quarantine	17 (25)	8 (4)	<0.001
Remained in your residence at all times, except for essential activities or exercise	48 (73)	177 (79)	0.28
Shared items like towels or utensils with other people	19 (28)	55 (25)	0.53
Had close contact (within 6 feet) with people who live with you	51 (76)	186 (83)	0.20
Had close contact (within 6 feet) with people who do not live with you	31 (46)	129 (58)	0.14
Gone outside to walk, hike, or exercise	33 (52)	196 (88)	<0.001
Shared a household with someone who has been interacting with more than 10 people/day	13 (19)	35 (16)	0.49
Used shared public transit (e.g. train, bus, or subway)	1 (1.5)	1 (0.4)	0.37
Used a ride-share service (e.g. uber or lyft) or taxi	3 (4)	1 (0.4)	0.04
Traveled by airplane	3 (4)	0 (0)	0.01
Worked or studied outside the home	27 (40)	56 (25)	0.02
Been exposed to 10 or more people per day through my work	24 (35)	40 (18)	0.002
Always worn gloves outside of home	6 (9)	18 (8)	0.24
Always washed my hands or used hand sanitizer after possible exposures	45 (67)	140 (63)	0.24
Always washed my hands for at least 20 seconds	50 (75)	165 (74)	0.60
Always cleaned and disinfected shared surfaces daily	34 (51)	94 (42)	0.51
Always cleaned or disinfected mail or groceries	9 (14)	51 (23)	0.18
Always avoided leaving my home at all	10 (15)	21 (10)	0.50
Always avoided others in public spaces	31 (46)	83 (37)	0.36
Always avoided touching my face while in public spaces	33 (49)	100 (45)	0.79
Always wore a mask	43 (64)	124 (55)	0.61
Which type of mask did you wear?			
Cloth	48 (71)	149 (66)	0.50
Surgical	27 (39)	80 (36)	0.53
N95	6 (9)	26 (12)	0.53
Other	2 (3)	9 (4)	0.69
Do you have any of the following health problems?			
Lung disease (e.g. asthma, chronic obstructive pulmonary disease)	10 (15)	29 (13)	0.70
Immune suppression	6 (9)	20 (9)	0.99
High blood pressure	18 (26)	70 (31)	0.47

## Discussion

In our study, only 40% of cases reported close contact with someone with COVID-19. Cases were more likely to report living with someone who had recent respiratory symptoms, possibly undetected infection. They were also more likely to provide direct patient care or live with someone who does. Healthcare workers are estimated to account for 3%-19% of COVID-19 cases in the United States [11]. These results reinforce the importance of testing and quarantining individuals with known or suspected exposure.

In our study, 60% of individuals reported no known contacts and were likely infected through community spread. One survey of infected patients found that most did not know the source of their infection, but over 80% worked outside the home in the two weeks preceding their diagnosis [12]. Our study adds to this by comparing cases to uninfected controls and confirming that work, study, or travel outside the home is a risk factor for SARS-CoV-2 infection. In contrast, controls were more likely than cases to go outside to walk or exercise, suggesting that outdoor exercise is relatively safe.

Overall, compliance with recommended social distancing was suboptimal. At the time of survey administration, there was low community spread in Ohio and Florida. About half the respondents had close contact with non-family members, only 60% always wore a mask, and two-thirds always washed their hands. At the same time, three-quarters remained in their homes except for essential activities, few attended gatherings of more than 10 people and almost none traveled by public transportation. We found no difference between cases and controls in the rates of event attendance, mask or glove usage, hand-washing, and cleaning surfaces/mail/groceries. However, this does not prove that these interventions are not protective.

Several limitations should be considered when interpreting the results of our study. Given that our survey was computer and telephone based, there may be non-response bias. Patients who responded may be more aware of and adherent to public health recommendations than the general public. Additionally, for some survey questions, the number of participants who answered 'yes' was low, potentially limiting our power to detect a smaller difference between the groups for those questions. Data were obtained via self-report and are subject to recall bias; however, given the short time frame (within the past seven days) this is less likely.

## Conclusions

It is important to identify which public health recommendations are most effective in limiting the spread of COVID-19 in order to support public adherence. Our study reveals the social behaviors and exposures of patients in Ohio and Florida from May to June 2020. The high percentage of infected individuals without known contacts or exposure to high-risk individuals reinforces the importance of contact tracing to reduce community spread, and social distancing. Infected individuals were more likely to have recently traveled and to work outside the home, emphasizing the importance of working remotely and minimizing travel.
